# Hepatocellular Expression of SIRT1 and Its Effect on Hepatocellular Carcinoma Progression: A Future Therapeutic Perspective

**DOI:** 10.1155/2020/2374615

**Published:** 2020-06-13

**Authors:** Meseret Derbew Molla, Gashaw Dessie, Yonas Akalu, Birhanu Ayelign

**Affiliations:** ^1^Department of Biochemistry, School of Medicine, College of Medicine and Health Sciences, University of Gondar, Gondar, Ethiopia; ^2^Department of Physiology, School of Medicine, College of Medicine and Health Sciences, University of Gondar, Gondar, Ethiopia; ^3^Department of Immunology and Molecular Biology, School of Biomedical and Laboratory Science, College of Medicine and Health Sciences, University of Gondar, Gondar, Ethiopia

## Abstract

Hepatocellular carcinoma (HCC) is an aggressive primary hepatic malignancy with a significant morbidity and mortality rate. Although chemotherapy along with surgical incision is believed to be an effective therapeutic approach, to date recurrence is being lifted a major concern. Thus, identifying another best therapeutic approach is becoming the main aim of physicians and scholars. In support of this, recently, several studies reported a significant observation of Sirtuin1 (SIRT1) overexpression in the malignant tumor cells, including HCC. As a result, they believed that overexpression of SIRT1 may have an effect on the progression of HCC by targeting growth and/or apoptotic controlling transcriptional factors/signaling pathways. Similarly, other reports confirmed that SIRT1 inhibition had a direct or indirect role in the control of tumor cell growth and metastasis. Therefore, inhibiting the expression and activity of SIRT1 might have a therapeutic effect to handle HCC. However, there are a limited number of reviews regarding the issue, and here, we summarized hepatocellular expression of SIRT1 and its role on HCC progression.

## 1. Introduction

Globally, liver cancer is one of the most common malignant tumors with an annual estimated death of 800,000 people [1]. In adults, HCC is the best known cause of hepatic malignancy bookkeeping for 70–85% of cases [2]. Even though researchers have revealed several predictors of HCC, chronic hepatic infections (HBV and HCV), alcoholic and nonalcoholic liver disease, aflatoxin B1, being male, and older age are the most common predictors of it [3]. In most cases, patients with HCC are asymptomatic until late stages, which makes it difficult to cure, since treatment outcome for advanced-stage HCC is very poor [4]. Therefore, addressing the molecular mechanism of tumor progression at early stage may aid for a successful treatment outcome. In support of this, several studies confirmed that there is a significant association between HCC progression and SIRT1 overexpression, and it is believed that controlling its overexpression may have a therapeutic role in HCC treatment.

Sirtuins are highly conserved protein-modifying enzymes first studied in the yeast silent information regulator 2 (Sir2) proteins, found from *Saccharomyces cerevisiae* in 1979 [5]. They located in distinct cellular compartments (nucleus, cytosol, or mitochondria) and coordinated the cellular responses to calorie restriction (CR) throughout the body system [6]. There are seven sirtuins (SIRT1-7) encoded in mammalian genomes. In mammals, SIRT1 (the homologue of the yeast Sir2 protein) is the best studied sirtuin. It is a nuclear nicotinamide adenine dinucleotide- (NAD^+^-) dependent protein deacetylase, responsible for regulating wide-ranging metabolic organs. SIRT1 yields a deacetylated protein, nicotinamide and 2-O-acetyl-ADP ribose by transferring the acetyl group of lysines in a protein substrate to the ADP-ribose moiety of NAD^+^ [7]. Despite having many substrates, its activity is tightly controlled by the intracellular levels of NAD^+^/NADH (nutrient availability) [8]. For the deacetylation of the target proteins, SIRT1 uses NAD^+^ as a common substrate [9]. SIRT1 is referred to as a master of the metabolic regulator; hence, it controls several transcriptional factors and cofactors involved in the metabolic homeostasis [10]. It is the best known molecular linker between the cellular energy status and adaptive transcriptional responses [9, 11]. SIRT1 can be expressed in the wide range of tissues and has been highly expressed in the liver, pancreas, heart, muscle, brain, and adipose tissue [12]. Furthermore, being at different subcellular sites (mainly the nucleus and cytoplasm and mitochondria) makes SIRT1 a major cellular energy sensor [8].

Several researchers reported that overexpression of histone deacetylases is observed in patients with cancers, thereby contributing to cellular transformation and tumorigenesis progression [13–15]. They also revealed that overexpression of SIRT1 is common in cancerous cells and had a potential effect to promote tumorigenesis. This may be due to SIRT1 controlling activity of the cell growth signaling pathway and/or regulation of SIRT1 target substrates in the cancerous cells. In support of this, reports confirmed that SIRT1 is involved in the progression of cancers by targeting the cell growth signaling pathways like Wnt-*β* and Akt/PI3K [16–18]. Actually, studies showed that SIRT1 controls the activity of the PI3K/AKT pathway by targeting its substrate called PTEN [13]. The PI3K/AKT pathway is an oncogenic pathway that inhibits apoptosis and enhances cell growth/survival and is negatively controlled by PTEN (tumor suppressor). With this coordination, SIRT1 promotes tumor cell survival and growth through the deacetylation and inactivation of PTEN, then indirectly activates the PI3K/AKT pathway [14, 15]. The role of SIRT1 in the Wnt/*β*-catenin signaling pathway in patients with HCC is not clearly addressed yet. However, one study reported that SIRT1 regulates the adipocyte and osteoblast differentiation via the induction of the Wnt/*β*-catenin signaling pathway in vivo and in vitro [16]. In addition, another study also revealed that SIRT1 activates the Wnt/*β*-catenin signaling pathways for the regulation of apoptosis and extracellular matrix degradation in osteoarthritis chondrocytes [17]. Based on these reports, we suggest that SIRT1 may have a direct/indirect role in tumor promotion/suppression.

The overexpression of SIRT1 can also directly inactivate p53 via deacetylation and further result in cell cycle control disturbance and upgrade tumor cells involved in cancerous cell progression [19, 20]. Likewise, other finding also showed that SIRT1 inhibition leads to the inhibition of cell proliferation and induction of cancer cell apoptosis [21]. Even though the role of SIRT1 in metabolism and life span is generally well defined, its activity in malignancy is complex, and either SIRT1 serves as a tumor silencer or a tumor promoter is even now a subject with level-headed discussion. Therefore, this review emphasized and summarized different research articles that showed hepatocellular expression of SIRT1 and its role on HCC progression.

## 2. Expression and Regulation of SIRT1 in Hepatic Cells at Physiological State

Initially, SIRT1 was identified as a histone deacetylase protein; however, right now it is also confirmed with nonhistone deacetylase target substrates [11]. The p53 was the first identified nonhistone substrate of SIRT1 and found to be deacetylated and repressed by it [10]. Soon after, other substrates, such as nuclear factor kappa B (NF-*κ*B), forkhead box O (FOXO) transcription factors, and peroxisome proliferator-activated receptor-*γ* coactivator 1*α* (PGC-1*α*), were revealed and control different cellular activities [12, 18]. Thus, transcriptional factors have a different cellular regulation activities governed by SIRT1. SIRT1 deacetylates these target proteins by consuming NAD^+^ as common raw material for all reactions, which is acetylated on specific lysine (Lys) residues to form deacetylated protein, NAM and OAADPR [9]. Then, the deacetylated protein can be stimulated or inhibited based on the regulatory nature of each substrate [12, 18].

Under physiological conditions, the activity of SIRT1 is regulated at many levels. It can be regulated by substrate availability, complex formation with other proteins, at level of gene expression, and posttranslational modification [19]. The NAD^+^ availability is the main determinant factor for SIRT1 activity; high intracellular NAD^+^ increases the activity of SIRT1 whereas low intracellular NAD^+^ level reduces SIRT1 activity [9]. The level of intracellular NAD^+^ becomes high during calorie restriction, prolonged fasting, or exercise [20]. This may be due to the activation of protein-kinase-A-mediated activation of the cyclic AMP (cAMP) response-element-binding protein by the glucagon [21]. In addition, polyphenols like resveratrol and quercetin can indirectly activate SIRT1. The polyphenols first activate the expression of AMP-activated kinase (AMPK), then NAMPT (enzyme responsible for NAD^+^ formation) for production of more NAD^+^ in the cells [22, 23]. The level of NAMPT will be further upregulated by AMPK, which will increase intracellular NAD^+^ and will further increase the level of SIRT1 [23].

SIRT1 expression can be also controlled by the nutritional availabilities and several transcription factors. High nutrition suppresses its activation, and conversely, low diet upregulates the expression of SIRT1 [24]. The transcriptional factors, such as cAMP response element-binding (CREB), peroxisome proliferator-activated receptors *α*&*β* (PPAR *α*&*β*), and FOXO1, upregulate the expression of SIRT1 while carbohydrate response element-binding protein (CHREBP) and PPAR*γ* downregulate the expression of SIRT1 [25–28]. Although the role of posttranslational modification of SIRT1 is not well documented, in an in vitro study, it is phosphorylated by cyclin-dependent kinase 1 (cyclin B–CDK1) complex and results in cell cycle disturbance [29]. Moreover, SIRT1 also sumoylated and suppressed following oxidative stresses like ultraviolet light exposure through enzymatic activity of sentrin-specific protease (SENP) [30]. Various researchers also revealed that SIRT1 can be regulated by complex formation with several other proteins. The complex formation of SIRT1 with active regulators of SIRT1 (AROS) upregulates its expression and activity [31]. Conversely, complex formation of SIRT1 with a silencing mediator of retinoids and thyroid hormone receptors (SMRT), nuclear receptor corepressor 1 (NCoR1), Lys-specific demethylase 1 (LSD1) and deleted in breast cancer 1 (DBC1) downregulate its activity [32–35].

## 3. Metabolic Effect of SIRT1 in Hepatic Cells

In mammals, including humans, the liver is a center of carbohydrate and lipid metabolism, which are termed as the principal energy fuels of humans [36]. Depending upon the physiological conditions, it maintains blood glucose and lipid level under normal conditions unless there are metabolic disorders. The liver cell is subjected to undergo glycogenolysis, gluconeogenesis, and ketogenesis through the recognition of low energy level in the cell (high NAD^+^/NADH ratio). Consequently, glycolysis, glycogenesis, lipogenesis, and protein biosynthesis become shutoff, and the reverse is true in the well-feeding state. Thus, activities are regulated and maintained by different hormonal and signal transduction pathways [36, 37]. It was also well established that mammalian sirtuins can have regulation activities with metabolic responses to nutritional input in multiple tissues/organs, including the liver [38]. In support of this, several evidences revealed that SIRT1 deacetylates transcription factors or enzymes involved in hepatic lipid and glucose metabolism [39, 40].

**Figure 1:**
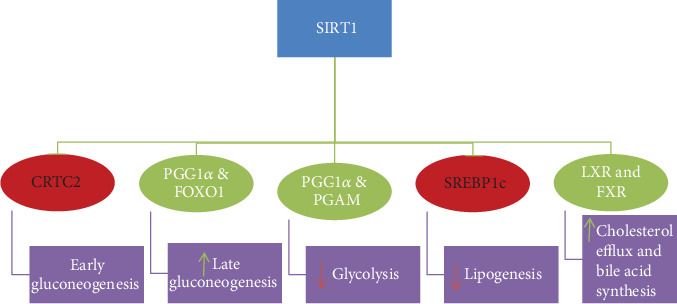
Metabolic role of SIRT1 in the liver. ∗SIRT1 deactivates CRTC2 and SREBP1c to decrease early gluconeogenesis and lipogenesis, respectively, while other regulatory transcriptional factors (PGC-1*α* and FOXO1, PGC-1*α* and PGAM, and LXR and FXR) are activated in the maintenance of blood glucose and lipid homeostasis. CRTC2: CREB-regulated transcription coactivator 2; SREBP1c: sterol regulatory element-binding protein 1c; PGC-1*α*: peroxisome proliferator-activated receptor-*γ* coactivator 1*α*; FOXO1: forkhead box O 1; LXR: liver X receptor; FXR: farnesoid X receptor.

Prolonged fasting or calorie restriction activates the expression of SIRT1, thereby stimulating PGC-1*α* and Foxo1 via deacetylation. PGC-1*α* and Foxo1 are involved in the maintenance of blood glucose level under normal condition and increase fatty acid oxidation [41, 42]. PGC-1*α* and Foxo1 activate gluconeogenesis (hepatic glucose production); the former also inhibits glycolysis simultaneously [41, 42]. In addition, SIRT1 inhibits glycolysis through direct suppression of the hypoxia inducible factor 1*α* (HIF1*α*) with deacetylation [43]. On the other hand, during early fasting state SIRT1 suppresses gluconeogenesis via CREB-regulated transcription coactivator 2 (CRTC2) deacetylation, resulting in CRTC2 deprivation and later decrement in transcription of gluconeogenic genes [44] (Figure 1).

SIRT1 also regulates lipid metabolism through the modification of liver X receptor (LXR), farnesoid X receptor (FXR), and sterol regulatory element binding protein (SREBP) [45–47]. The SREBP promotes the expression of lipogenic and cholesterolgenic genes to regulate lipid metabolism whereas liver X receptor and farnesoid X receptor are the best known receptors by their regulatory activity on cholesterol and bile acid metabolism [48, 49]. SIRT1 controls the activity of LXR through deacetylation, ubiquitination, and turnover of LXR and so improves the expression of ATP-binding cassette transporter A1 (ABCA1), which is an LXR target gene, to promote cholesterol efflux from cells [45]. SIRT1 also inactivates SREBPs via deacetylation after fat and cholesterol synthesis is turned off [47] (Figure 1).

## 4. Role of SIRT1 in Hepatocellular Carcinoma Progression

The role of SIRT1 in tumorigenesis development and progression is not clearly defined to date. This is due to the controversy of published articles that reported SIRT1 regulates different transcriptional factors acting as a tumor suppressor and/or a tumor promoter protein [50]. An overexpression of SIRT1 had been recognized in several tumor cells, including HCCs. This may be due to the realities by which SIRT1 activity manages signaling pathways connected with cell growth and motility [51–53]. Its overexpression might need grave results for tumor progression. In vitro and in vivo models by Portmann et al. stated that SIRT1 is sturdily overexpressed in patients with HCC. They also reported that inhibiting the activity of SIRT1 in HCC had a cytostatic effect in vitro, which is characterized by impaired cell proliferation, and results in impaired tumor cell growth in vivo [54].

In line with this, another study also reported that overexpression of SIRT1 in HCC cells is positively associated with micro vascular invasion and metastasis through the stimulation of PGC-1*α*-mediated mitochondrial biogenesis resulting in poor treatment outcome [55]. This was also supported by Frazzi, who showed that an overexpression of SIRT1 results in poor treatment outcome and short life expectancy in patients with HCC [56]. But be sure that the subcellular localization of SIRT1 can be varied from cell to cell, and some cells appeared to have nuclear expression of SIRT1 whereas others expressed it within the cytoplasm or in both [57]. Reports stated that cytoplasmic SIRT1 is overexpressed in cancerous cells, but did not mention its clinical significance in those cells [58, 59]. Prominently, one study reported that nuclear SIRT1 promotes HCC progression whereas cytoplasmic SIRT1 suppresses the progression of it [60]. They aimed to detect the presence or absence of SIRT1 overexpression and its clinical significance in patients with HCC and found a statistically significant overexpression of SIRT1 in HCC tumor tissues compared with adjacent nontumor tissues. The overexpression of SIRT1 was observed in both the nucleus and the cytoplasm. Along with this, they also found a significant association of nuclear SIRT1 overexpression with overexpression of target substrates, such as P53 and YAP2. The author(s) have also done survival and multivariate analysis and found that the overexpression of nuclear SIRT1 was associated with poor overall survival and independent tumor promoters whereas the survival and multivariate analysis result of cytoplasmic SIRT1 overexpression was found to be a predictor of longer overall survival and an independent tumor suppressor [60].

However, either SIRT1 acts similarly as a tumor promoter or tumor silencer remains dubious. The reason behind is pointed out by four variables. (i) SIRT1 can be overexpressed or underexpressed in different sorts of human cancers. For example, SIRT1 overexpression was reported in gastric, colon, ovarian, and breast cancer cells while it was underexpressed in glioblastoma and bladder cancer [61–65]. (ii) SIRT1 represses those outflows or action in a significant number of tumor suppressors and oncoproteins. Several reports confirmed that the overexpression of SIRT1 represses the expression and/or activity of different tumor suppressor genes and proteins, such as SFRP1, SFRP2, GATA4, GATA5, CDH1, MLH1, NBS1, WRN, and FOXO family members (FOXO1, FOXO3a, and FOXO4) [66–69]. In addition, published papers mentioned that overexpression of SIRT1 deacetylates and inactivates p53, which is the well-known gene for the regulation of apoptosis [70]. (iii) SIRT1 induces growth capture because of the opposition with DNA damage. One experimental study revealed that upregulation of SIRT1 modulates p53 activity in DNA damage response and apoptosis through hypermethylated in cancer 1- (HIC1-) SIRT1-p53-HIC1 loop pathway. This pathway initiates with a complex formation of HIC1 and SIRT1, which is a transcriptional repression complex; activation of HIC1 downregulates SIRT1 or the activation of the former transcriptional factor upregulates the latter one. As SIRT1 activates due to inactivation of HIC1, then the p53 gene becomes inactivated, which will further result in the lack of apoptosis activation and survival of damaged DNA [70]. The HIC1 encodes a transcriptional repressor with zinc finger motifs, and its role in the regulation of proliferation and p53-dependent survival in several tumors through DNA methylation is reported [71, 72]. (iv) SIRT1 could both enhance the lifespan and diminish those hazards from claiming cancer [73, 74]. In support of this, one study reported that SIRT1-deficient mouse embryonic fibroblasts (MEFs) have an increased resistance to replicative senescence, and under conditions of chronic and sublethal oxidative stress, these mice were correlated with extended replicative life span. The author(s) then predicted that SIRT1-deficient cells are unable to regulate either the senescence regulator or p53 [75]. From here, we generalized that SIRT1 may have a protective activity on tumor initiation under normal cells although once developed it may facilitate tumor progress. Other studies also reported that SIRT1 prevents tumor initiation through the promotion of telomere maintenance, the activity of homologous recombination and DNA repair [76, 77].

**Figure 2:**
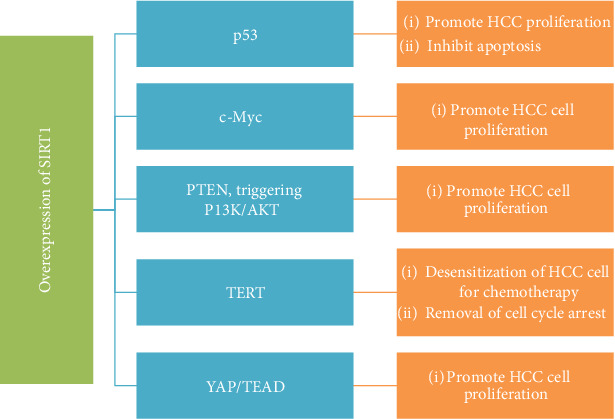
Effect of SIRT1 in HCC cell progression. ∗SIRT1 deacetylates p53 and PTEN and results in the promotion of HCC cell proliferation. The later transcription factor is through the inactivation of the P13K/AKT signaling pathway. On the other hand, SIRT1 activates oncogenes (c-Myc, TERT, and YAP/TEAD) and is further involved in HCC cell progression. HCC: hepatocellular carcinoma; PI3K: phosphatidylinositol 3 kinase; AKT: protein kinase B; c-Myc: cellular homologue of avian myelocytomatosis virus oncogene; PTEN: phosphatase and tensin homologue deleted on chromosome 10; YAP: yes-associated protein; TEAD: TEA domain family member 4.

With these controversies, researchers showed that SIRT1 might have essentially lifted in the HCC tissues when compared with that of nontumor tissues [15, 78]. The SIRT1 advertises tumorigenesis of HCC through PTEN/PI3K/AKT indicating pathway [15]. Through the help of RNA interference, they checked the overexpression and selective silencing of SIRT1 in hepatoma and normal cell lines and found a significantly higher overexpression of SIRT1 in hepatoma cell lines compared with normal cell lines. The author(s) further assessed the downregulating effect of SIRT1 in HCC cell induction on apoptosis and reported that downregulation of SIRT1 stimulates apoptosis. Finally, they reported that SIRT1 silencing-induced apoptosis is PI3K/PTEN/AKT signaling pathway-dependent. Thus, when SIRT1 was inhibited, AKT phosphorylation was significantly increased and the level of PI3K increased whereas the upregulation of SIRT1 could have an opposite effect [15]. The PI3K/AKT pathway is a vital oncogenic pathway that upholds cell growth and existence [14]. Thus, the pathway is negatively controlled by PTEN, which is a target of SIRT1 [13]. The SIRT1 deacetylates PTEN and inactivates the PI3K/AKT pathway. Furthermore, inhibition of SIRT1 activates AKT and PI3K via phosphorylation [15] (Figure 2). On the other way, SIRT1 and c-Myc can work synergistically to stimulate hepatocellular proliferation in HCC cells and adjust each other through a positive feedback way, which results in poor treatment outcome [79] (Figure 2). c-Myc is one of the main oncogenes for the initiation of HCC tumors, and an experimental study in mice reported that inactivation of c-Myc suppresses liver tumors [80]. The overexpression of SIRT1 can also directly inactivate p53 or activate the oncogene; c-Myc during tumorigenesis implies a predominant oncogene role of SIRT1 [81, 82].

Another study also reported that overexpression of SIRT1 promotes HCC growth progression via telomere maintenance [51]. The study stated that an overexpression of SIRT1 is found in HCC cell lines compared to the controls, and there was a statistical association between advancement of the tumor grade and expression of SIRT1. Tissue microarray analysis of HCC and adjacent nontumoral liver tissues revealed a positive correlation between the expression levels of SIRT1 and advancement of tumor grades. They also reported that silencing of SIRT1 consistently inhibited the proliferation of HCC cells through the stimulation of apoptosis and/or inhibition of telomerase reverse transcriptase (TERT) expressions [51]. Therefore, SIRT1 might do likewise a novel target for specific slaughtering of tumor versus nontumor liver cells.

## 5. Therapeutic Effect of SIRT1 Inhibition in HCC Treatment

As stated earlier, SIRT1 overexpresses in various tumors, including HCC [54, 83]. It was also reported that inhibition of SIRT1 can have a positive effect on the reduction of the prostate cancer cell growth [84]. The author(s) tried to check the effect of SIRT1 overexpression or inhibition in the growth and viability of human prostate cancer, and they found that overexpression of SIRT1 was statistically correlated with the prostate cancer cell overgrowth. The author (s) also reported that in prostate cancer, inhibiting SIRT1 results in an increment of FOXO1 activation. Based on these finding, they finally generalized that SIRT1 inhibition can help on the treatment of prostate cancer through the activation of FOXO1, and overexpression of SIRT1 could have an opposite effect [84]. It is also possible that the reduction of prostate cancer after inhibition of SIRT1 could be due to compensatory overexpression of other sirtuins that have demonstrated tumor suppressor effects, such as SIRT3 and SIRT6. Thus, sirtuins have the ability to promote tumor suppression through the inhibition of tumor-promoting transcription factor expression, such as hypoxia-inducible factor 1-alpha (HIF-1*α*), NF-*κ*B, c-Myc, nuclear pyruvate kinase M2 (PKM2), and Twist1 [85–88]. In addition, SIRT3 and SIRT6 help to suppress tumor cell growth through the activation of tumor suppressor transcriptional factor (FOXO3A) by inhibiting the Wnt/*β*-catenin pathway and by activating the antiapoptotic activity of survivin [89, 90]. In prostate and bladder cancer, inhibition of SIRT6 due to the hyper expression of E2F transcription factor 1 (E2F-1), which is a target of SIRT1, promotes the progression of cancer cell overgrowth and results in poor prognosis of the disease. In other word, the inhibition of SIRT1 suppresses the expression of E2F-1; consequently, it activates SIRT6, which reduces the progression of cancers [91, 92]. Therefore, inhibiting the activity of SIRT1 may have a potential effect for suppression of the tumor cells growth, and this may be a new means of HCC treatment approach.

**Figure 3:**
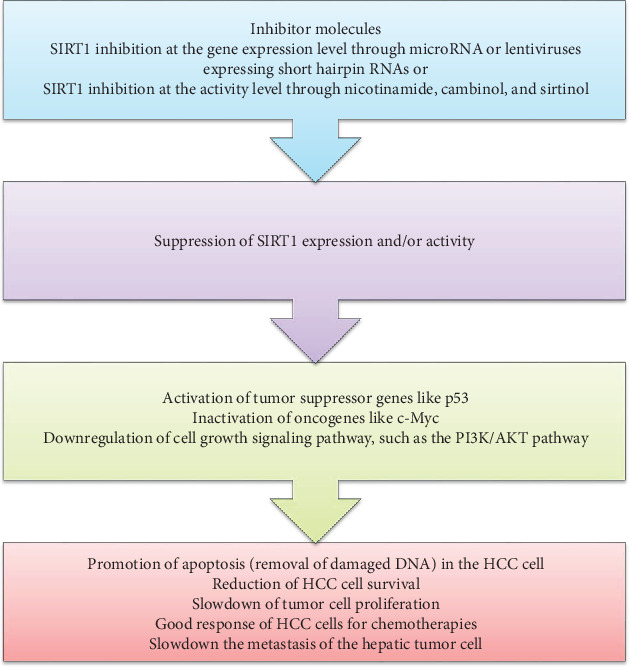
The therapeutic role of SIRT1 inhibition in HCC treatment. ∗Inhibition of SIRT1 by different small molecules results in the activation and inactivation of tumor suppressor and oncogenes, respectively. This further results in a good chemotherapeutic treatment outcome of the disease, slowdown of the progression, and stimulation of apoptosis of HCC cells.

To date, the therapeutic approach of HCC is not effective as expected, especially in developing countries due to the diagnosis of the disease at late stage. Chemotherapy along with surgical incision is believed to be effective but not curative because of recurrence [93–95]. Therefore, considering other best option is required from researchers. As we understand from the earlier discussions, SIRT1 is involved in tumor cell growth and progression immediately when it transforms into malignancy. Because of this, scholars are interested in justifying the effect of SIRT1 inhibition on the suppression of the tumor cell proliferation. Furthermore, the positive impact of SIRT1 inhibition for improvement of cancers through the stimulation of apoptosis and inhibition of SIRT1 target oncogenic genes is reported [84, 96]. The inhibition of SIRT1 reduces the expression and/or activity of several oncogenes, such as c-Myc and PI3K/AKT pathway. Interestingly, a study indicated that inhibition of SIRT1 prepares HCC cells to manage it with doxorubicin [51]. Another researcher also showed that upregulation of SIRT1 has a strong association with HCC cell growth progression and promotes chemotherapy resistance for these patients [63]. In addition, SIRT1 inhibition via microRNA-133b slows down HCC progression [97] (Figure 3). MicroRNAs (miRNAs) are a small noncoding group of RNAs that act as gene regulators to regulate the expression of messenger RNAs (mRNAs), then results in translational repression [98]. In vitro, overexpression of mRNA-133b increases tumor cell death rather than reduces proliferation [97].

Similarly, another study also reported that overexpression of SIRT1 in human HCC cells and knockdown of its activity result in the increment of differentiation marker expression and impairment of cancer cell progression in in vitro and in vivo models [54]. They also revealed that in an orthotopic xenograft model, inhibition of SIRT1 has a potential to reduce the progression of tumor cell growth and a good response rate when treated with chemotherapies [54]. The activity of SIRT1 can be inhibited by lentiviruses expressing short hairpin RNAs or with a small molecule inhibitor like cambinol [52]. The roles of these inhibitors are to silence the activity of SIRT1 in tumor cells, then to identify the effect of these cells in comparison with cells with overexpression of SIRT1. As we have discussed earlier, overexpression of SIRT1 targets growth-controlling transcription factors, such as p53, c-Myc, and FOXO, and results in a direct effect on cancer cell survival and proliferation. Consequently, it also inhibits cellular senescence and differentiation to promote cancer cell growth. Therefore, SIRT1 inhibition is believed to be effective for cancer therapy through the induction of cancer cell apoptosis and/or suppression of cancer cell proliferation. This is due to the fact that inhibition of SIRT1 upregulates tumor suppressor signals and downregulates growth-promoting signals and puts the cells in an apoptotic and nonproliferating state [54, 99] (Figure 3). The overexpression of SIRT1 has also a strong association with invasion and metastasis of HCC via epithelial-mesenchymal transition, which results in poor treatment outcome [100]. In addition, overexpression of SIRT1 in HCC showed cisplatin (hepatoma cell-sensitive chemotherapeutics) treatment resistance [101] (Figure 3). Furthermore, inhibition of SIRT1 has a positive irradiation efficacy in the treatment of hepatic tumor cells [102]. Therefore, considering SIRT1 inhibition as a therapeutic alternative for HCC should be encouraged and may be taken as one of the best approaches to cure HCC. However, further extensive studies on the association of SIRT1 and HCC progression are mandatory.

## 6. Conclusion and Future Perspectives

Under normal conditions, SIRT1 can have regulatory activities in liver cells to maintain energy metabolic hemostasis; it can even bring out against tumor cell initiation through the deacetylation and activation of tumor suppressor genes. However, once the tumor cell initiated, it can aggravate the progression by manipulating several oncogenic transcriptional factors, including the PI3K/AKT pathways. Therefore, inhibiting the activity of SIRT1 has a protective role to limit HCC progression. Hopefully, in the near future, many researchers will address the exact molecular mechanism of SIRT1 inhibition and its effect on HCC treatment, and it will be one means of approach to manage the worst aspects of HCC.

## Abbreviations

HCC: Hepatocellular carcinoma
NAD^+^: Oxidized nicotinamide dinucleotide
SIRT: Sirtuins
SIR2: Silent information regulator 2
CRTC2: CREB-regulated transcription coactivator 2
SREBP1c: Sterol regulatory element-binding protein 1c
PGC-1*α*: Peroxisome proliferator-activated receptor-*γ* coactivator 1*α*
FOXO1: Forkhead box O 1
LXR: Liver X receptor
FXR: Farnesoid X receptor.
